# A graphical, interactive and GPU-enabled workflow to process long-read sequencing data

**DOI:** 10.1186/s12864-021-07927-1

**Published:** 2021-08-23

**Authors:** Shishir Reddy, Ling-Hong Hung, Olga Sala-Torra, Jerald P. Radich, Cecilia CS Yeung, Ka Yee Yeung

**Affiliations:** 1 University of California, 92697 Irvine, CA USA; 2 School of Engineering and Technology, University of Washington, 98402 Tacoma, WA USA; 3 Clinical Research Division, Fred Hutchinson Cancer Research Center, 98109 Seattle, WA USA; 4 Clinical Research Division, Kurt Enslein Endowed Chair, Fred Hutchinson Cancer Research Center, 98109 Seattle, WA USA; 5 Department of Medicine, University of Washington, 98109 Seattle, WA USA; 6 Department of Laboratory Medicine and Pathology, University of Washington, 98109 Seattle, WA USA

**Keywords:** Cancer diagnostics, Workflows, Cloud computing, Nanopore, GPU, FAIR, Long-read sequencing, Leukemia

## Abstract

**Background:**

Long-read sequencing has great promise in enabling portable, rapid molecular-assisted cancer diagnoses. A key challenge in democratizing long-read sequencing technology in the biomedical and clinical community is the lack of *graphical* bioinformatics software tools which can efficiently process the raw nanopore reads, support graphical output and *interactive* visualizations for interpretations of results. Another obstacle is that high performance software tools for long-read sequencing data analyses often leverage graphics processing units (GPU), which is challenging and time-consuming to configure, especially on the cloud.

**Results:**

We present a graphical cloud-enabled workflow for fast, interactive analysis of nanopore sequencing data using GPUs. Users customize parameters, monitor execution and visualize results through an accessible graphical interface. The workflow and its components are completely containerized to ensure reproducibility and facilitate installation of the GPU-enabled software. We also provide an Amazon Machine Image (AMI) with all software and drivers pre-installed for GPU computing on the cloud. Most importantly, we demonstrate the potential of applying our software tools to reduce the turnaround time of cancer diagnostics by generating blood cancer (NB4, K562, ME1, 238 MV4;11) cell line Nanopore data using the Flongle adapter. We observe a 29x speedup and a 93x reduction in costs for the rate-limiting basecalling step in the analysis of blood cancer cell line data.

**Conclusions:**

Our interactive and efficient software tools will make analyses of Nanopore data using GPU and cloud computing accessible to biomedical and clinical scientists, thus facilitating the adoption of cost effective, fast, portable and real-time long-read sequencing.

**Supplementary Information:**

The online version contains supplementary material available at 10.1186/s12864-021-07927-1.

## Background

Cancer is a leading cause of mortality worldwide with the highest burden of death affecting lower- and middle-income countries [1]. Delays in medical care from the inability to detect cancer earlier are a key component contributing to higher morbidity, poor response to treatments and lower survival [2]. Advances in molecular diagnosis have enabled detection of specific driver mutations that can be essential for prognosis, monitoring, and targeted therapy [3–5]. Examples of such “precision medicine” include the BCR-ABL fusion gene in chronic myeloid leukemia (CML), PML-RARA fusions in acute promyelocytic leukemia (APL) and FLT3 mutations in acute myeloid leukemia (AML) [6–9]. Potentially targetable mutations are also found in solid tumors, such as renal cell carcinoma [10–12]. Currently, detection of fusion genes by chromosomal analysis requires highly specialized laboratories. Chromosomal analyses may not have the precision necessary to identify the specific breakpoint in a patient or provide the sequence of the flanking segments around the fusion gene to allow for downstream development of patient specific monitoring assays. Routine PCR based assays can be rapid, but a priori knowledge of the fusion breakpoints is required and when fusions involve large intronic regions RNA input is generally needed. Thus, turn-around times for these methods can take three days to two weeks [13, 14]. To capitalize on the potential of precision medicine, faster analysis of sequencing data is needed to improve the potential of molecular-assisted cancer diagnoses [15].

For cancer management, next generation sequencing (NGS) has many limitations, such as phasing errors, mis-mapping from short reads, strand bias and amplification errors causing irregular variant allele frequency, and bioinformatically-challenging repetitive sequences [16, 17]. In contrast, long-read sequencing technology, such as Oxford Nanopore Technologies (ONT), generates continuous sequences up to a few megabases in length at this time [18]. Nanopore sequencing provides both sequencing and phasing information because the read lengths are generally very long in comparison to NGS (NGS = 150 to 250 bp, nanopore = generally > 2 kb to 200,000 kb or longer), therefore it will not incur the same artifacts and mapping errors [19]. Unlike NGS, which takes an average of three days to complete sample processing and library preparation plus one additional day for sequencing, nanopore sequencing can directly sequence DNA, resulting in much shorter turnaround times [20, 21]. Thus, long-read sequencing technologies hold promise in overcoming the current diagnostic gap in cancer research.

Computational methods and software tools tailored for long-read sequencing data are essential to enable use of this emerging and promising technology [22, 23]. In nanopore sequencing, electrical current alterations are recorded as different bases traverse the pore opening. Basecalling, which translates the signal (stored as fast5 files) into a sequence of base pairs is the key step determining accuracy of the sequencing experiment. The initial conversion is followed by error correction and data polishing to obtain the final sequence [23]. Basecalling is computationally expensive and a rate-limiting step in the analysis of nanopore data. In contrast, for NGS data, aligning reads is the rate limiting step due to the greater number of reads and the absence of a separate basecalling step. Deep learning neural network models have been applied to basecalling to increase the accuracy [24]. With standard CPU processing, these methods are prohibitively slow and require large numbers of computational cores operating in parallel to be practical. Graphics processing units (GPUs) can be used to accelerate the analysis but require specialized hardware and software. Hardware in the form of GPU instances are available on public cloud services such as Amazon Web Services (AWS). However, virtual machine instances do not come with the drivers or Compute Unified Device Architecture (CUDA) libraries installed. In addition, the versions of these drivers and libraries must be carefully matched to the software being executed.

Nanopore sequencing is fast and cost effective in terms of data collection, with a $90 USB Flongle attached to a laptop supporting real-time sequencing. Sequencing is potentially accessible to a broad range of biomedical scientists who do not have access to a traditional sequencer. A challenge in democratizing long-read sequencing technology is the difficulty of the processing of the data which requires command line tools and technically difficult installation of software, libraries and drivers. There is a lack of *graphical* bioinformatics software tools which can efficiently process the raw nanopore reads, and *interactive* visualizations for interpretations of results. An example of a command-line workflow is the MasterOfPores pipeline that performs pre-processing and analysis (prediction of RNA modifications and estimation of polyA tail lengths) of long-read data [25]. MasterOfPores is a workflow using the NextFlow framework [26], a script-based engine that requires programming experience to deploy and modify. While MasterOfPores uses software containers for most of its components, it does not provide a container for the key basecalling step which requires the most setup and configuration to operate with GPU computing. In addition, MasterOfPores does not include the product-grade basecaller Guppy [27], which is available to ONT customers via their community site [28] and cannot be distributed in a container.

## Implementation

**Fig. 1:**
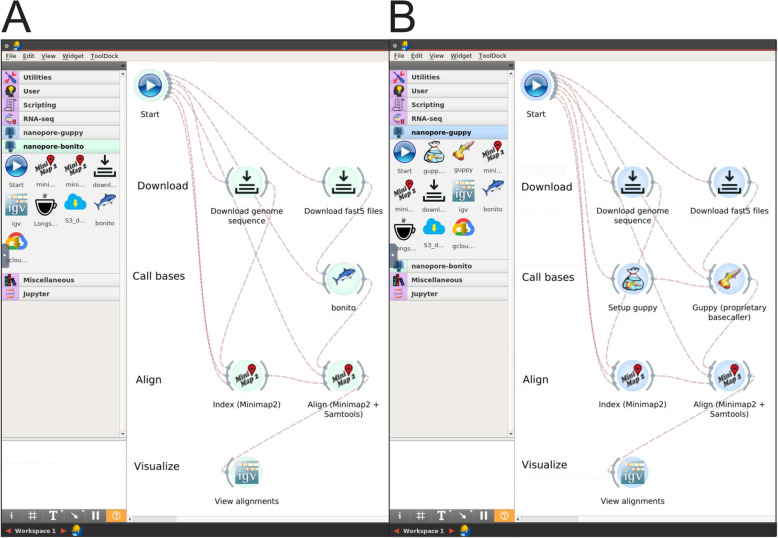
Screenshots of our interactive GPU workflow which uses the Biodepot-workflow-builder platform. Panel A is a screenshot of the workflow using the open-source Bonito basecaller. Panel B is a screenshot of the workflow using the proprietary Guppy basecaller. Both basecallers use GPUs. For the Guppy workflow, the user enters the URL for the Oxford Nanopore Technology Guppy installation package which is then used to create a container to execute Guppy. The other steps in the two workflows are identical, consisting of data download, alignment and visualization. Each of these steps are performed by software modules encapsulated in Docker containers and represented by the graphical widgets. Lines connecting the widgets indicate flow of data between the modules. The user double clicks on the Start widget, enters the necessary parameters into the forms and presses a graphical start button to start the workflow. Double-clicking on a widget brings up a point-and-click interface for users to enter parameters, monitor results and control execution of the associated workflow module. Unlike other workflow execution platforms, the Biodepot-workflow-builder supports modules with interactive graphics. This is leveraged in this workflow to automatically open the final BAM files in the Interactive Graphics Viewer (IGV) which we use to check for diagnostic translocation breakpoints in our cell-line data. The execution time of the basecallers Guppy and Bonito on GPU-enabled machines using the NB4 cell line averaged 88.9 s (standard error 1.2) and 948.2 s (standard error 1.7) on an AWS g4dn.4xlarge GPU instance. For comparison, the CPU version of Guppy averaged 2551.8 s (standard error 22.4) on an AWS virtual machine instance (c5d.18xlarge) using 72 vCPUs

We present a graphical cloud-enabled containerized workflow for fast, interactive analysis of nanopore data using GPUs. Specifically, we extended the Biodepot-workflow-builder (Bwb) [29] to provide a modular and easy-to-use graphical interface that allows users to create, customize, execute, and monitor bioinformatics workflows. Figure 1 shows screenshots of the platform. The workflow consists of modules to download the data and genome files, basecallers Guppy [27] or Bonito [30], minimap2 [31] for sequence alignment, and the Integrated Genome Viewer (IGV) [32, 33] for visualization of the BAM files. We provide containers for both the ONT proprietary Guppy [27] and the ONT open-source Bonito [30] basecallers. In accordance with the licensing constraints of Guppy, we provide a containerized setup module that creates the Guppy container locally when the user provides the download URL from the ONT community site. To facilitate deployment, we use Docker containers for all modules (including the Bwb platform) and provide an Amazon Machine Image (AMI) with all software and drivers pre-installed for GPU computing on the cloud.

### Integrated and interactive workflow in the Biodepot-workflow-builder (Bwb)

Biodepot-workflow-builder (Bwb) [29] provides a modular and easy-to-use graphical interface for reproducible execution, customization and interactive visualization of the nanopore pipeline. Graphical widgets representing Docker containers that execute modular tasks are graphically linked to define bioinformatics workflows that can then be reproducibly deployed across different local and cloud platforms. New widgets (modules) can be added without writing code. In this work, we created new widgets in the Bwb for basecalling using Guppy [27] and Bonito [30], alignment using minimap2 [31], visualization of the resulting BAM files using the Integrated Genome Viewer (IGV) [32, 33]. Each of these widgets call a Docker container in the backend. Users can adjust input parameters of each widget using an intuitive form-based user interface, and check intermediate results using a console. A key characteristic of Bwb is integrated support for graphical output, enabling *interactive* tools such as Jupyter notebooks, spreadsheets, and visualization tools to be included in the workflow. We leverage this graphical output support feature of the Bwb to create an integrated, interactive workflow by integrating the IGV to visualize resulting BAM files. Our workflows can be shared using a Bwb native format or exported as shell scripts. In addition, the Bwb is distributed as a Docker container that can be easily deployed on *any local or cloud platform*.

### GPU software versioning and compatibility ensured by using containers and providing an AMI with drivers and software

GPU-enabled executables require additional software layers to use the GPU hardware. For NVIDIA hardware, CUDA software provided by the manufacturer is necessary to perform general computations on the GPU. AMD GPUs use a different and incompatible set of software for their hardware. In addition, low level libraries (drivers) are required to communicate with the GPU card. Additional language and operating system dependent libraries and headers may also be required to integrate the CUDA software. All these layers of software interact with each other and as a result, compatibility is version dependent and even sensitive to the method of installation. Components installed using scripts may be incompatible with components installed using package managers.

An example of the complexities involved in deploying GPU software is the open-source Bonito basecaller. The current version of the Bonito caller will not install if one follows the instructions on the Github [30]. This is due to dependency incompatibilities in the current Python PyTorch [34] packages and CuPy [35] libraries. We were able to install Bonito v0.38 by downgrading from CUDA 11.3 to CUDA 10.2, cuDNN 7, CuPy 10.2. and downgrading PyTorch from 1.8.0 to 1.7.1. By providing a Docker container we can ensure that Bonito is deployed in this compatible environment without the need for the user to install the exact versions of the software. However, this is not sufficient, the user still needs to install the compatible drivers and CUDA software on their host machine. This is true even for GPU instances on Amazon cloud. AWS does provide the basic Linux operating systems but requires that users install their own drivers and libraries. There are commercial distributions on the AWS marketplace that provide support, but we could find nothing among the free community offerings. As a resource for the nanopore research community, we therefore have provided a public freely available AMI for use with AWS GPU virtual machine instances with versions of drivers and libraries that we have tested with our workflows. The combination of AMI and containerized modules eliminates the arcane installation steps and makes the nanopore GPU software accessible to a broad audience in the biomedical and clinical community.

## Results

### Data generation using cell lines

5,000 ng of DNA from four cell lines (NB4, K562, ME1, MV4;11) was dephosphorylated and cut with Cas9 enzyme complexed with RNA guides designed to target the genes involved in the translocations present in each line (BCR and ABL1 for K562, PML and RARA for NB4, CBFB and MYH11 for ME1, KMT2A and AFF1 for MV4;11). Nanopore adapters were then ligated to the newly created DNA ends and the library was loaded onto a flow cell or Flongle and sequenced using a MinION Mk1B.

### Detection of fusion genes in blood cancer cell lines

**Fig. 2:**
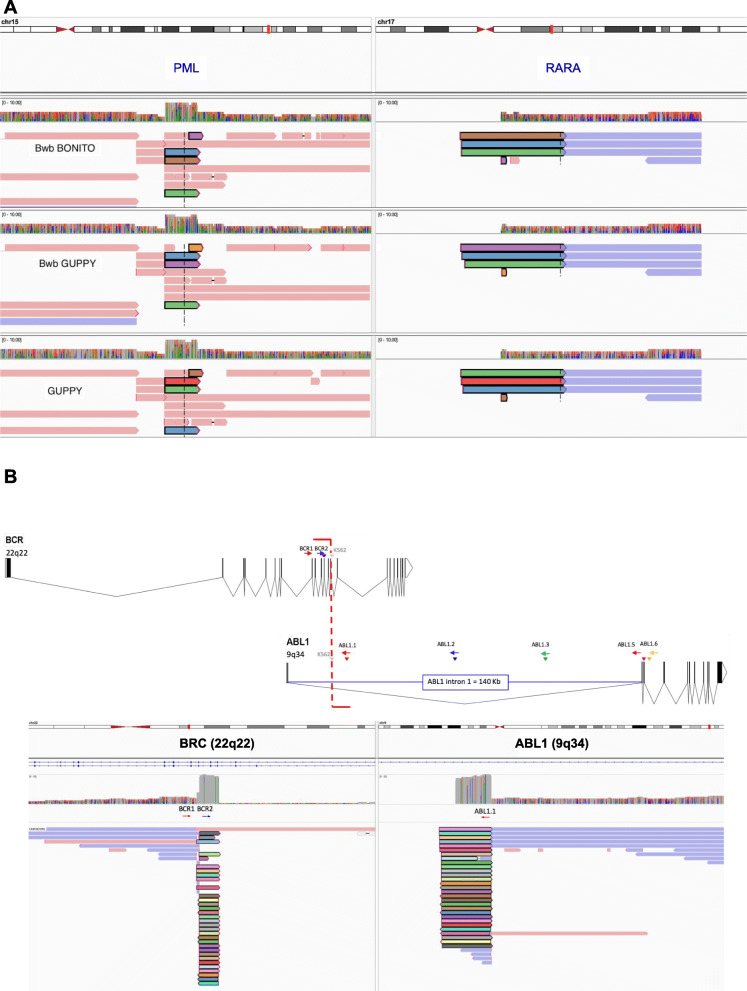
**A**. IGV viewer alignment on the PML and RARA genes of a Flongle Nanopore generated sequences for the NB4 cell line. Library was generated from DNA with a PCR-free enrichment protocol using CRISPR guides targeting PML and RARA genes. The top panel shows the alignment for the reads processed with the Bonito basecaller and minimap2 aligner in the Bwb. The middle panel shows the alignment of reads processed with the Guppy basecaller and minimap2 aligner in the Bwb workflow. The bottom panel shows reads processed in a manual step-by-step workflow using the Guppy flipflop basecaller and minimap2 aligner. Reads with PML-RARA breakpoint are colored to highlight the fragment aligned to PML and RARA.**B**. Genomic BCR-ABL1 breakpoint identified in the K562 cell line by long-read sequencing. Schematic representation (generated with http://wormweb.org/exonintron) shows the breakpoint captured with our amplification-free enrichment protocol and long-read sequencing. The breakpoint is represented in the upper graphic by the red vertical line, and the location of the sequence specific guides is marked by colored arrows. ABL1 intron 1 spans 140Kbs. In the lower panel, nanopore sequence alignments in IGV show sequences partially aligned to BCR and ABL1. Reads with BCR-ABL1 breakpoint are colored to highlight the same read is partially aligned to BCR and ABL1.

Applications of nanopore sequencing coupled with our workflows in cancer diagnostics are shown in Fig. 2. We can reliably detect fusion genes from DNA sequences using cell lines (NB4, K562, ME1, and MV411) with known fusion genes. This provides an advancement to molecular diagnostics by its ability to detect specific breakpoints even if there is a large intronic region between the fusion genes of interest. DNA sequencing of NB4 on a comparatively low cost ($90) Flongle nanopore device, (Oxford, UK), confirmed fusion gene sequencing spanning the *PML* and *RARA* genes (Fig. 2a). DNA sequencing of K562 on a Flongle was able to capture the fusion gene spanning the *BCR* and *ABL1* genes, as well as capturing the intervening large *ABL1* intronic region 1 where the specific breakpoint occurs (Fig. 2b). Figure S1 in Additional file 1 presents additional results for the detection of fusion genes CBFB and MYH11 for the cell line ME1 (Figure S1A), KMT2A and AFF1 for MV4;11 (Figure S1B).

### Benchmarking basecallers

We compared the execution time of the basecallers Guppy [27] and Bonito [30] on GPU-enabled machines using the NB4 cell line data. We also measured the execution time of running Guppy using CPU and on a local host. The runtime of Guppy is reduced from over 42 min to just over 1 min using GPU computing, representing a *29x speedup and a 93x reduction in cloud computing costs*. The results are summarized in Table 1.

**Table 1:** Comparison of runtime from different basecallers (Guppy and Bonito) using the NB4 cell line. The AWS results were averaged over 4 runs. The local host results were averaged over 5 runs

Basecaller	cloud/local	average runtime (seconds)	standard error (seconds)
Guppy CPU	AWS c5d18xlarge	2551.8	22.4
Guppy GPU	AWS g4dn.4xlarge	88.9	1.2
Guppy GPU	Laptop	135.3	0.6
Bonito GPU	AWS g4dn.4xlarge	948.2	1.7

### Experimental setup for benchmarking

Guppy GPU was benchmarked on an AWS g4dn.4xlarge virtual machine instance with a NVIDIA Tesla T4 GPU with the template_r9.4.1_450bps_hac.jsn model file provided by ONT. Bonito GPU was also benchmarked on the same instance using the provided dna_r9.4.1 model file and the default settings (chunk size of 4000 and batch size of 32). Guppy CPU was benchmarked on a c5d18xlarge instance with 72 vCPUs, 72 threads/basecaller, and 1 basecaller. The Guppy GPU experiments on a local host were performed on a laptop with a GeForce RTX 2060 GPU. All benchmark experiments on AWS were based on 4 runs. We observed that Guppy GPU achieved the fastest average time at 88.9 s (1.5 min) with standard error of 1.2 s. Bonito GPU finished basecalling in 948.2 s (15.8 min) on average with standard error of 1.7 s. Guppy CPU achieved the slowest average time at 2551.8 s (42.5 min) with standard error of 22.4 s. The 29x speedup is computed by comparing the average runtime (in seconds) of Guppy CPU to Guppy GPU *(2551.8/88.9 = 28.7).*

### Estimation of costs of basecalling step

A large selection of virtual machine instance types with different pricing structures are available on AWS [36]. We conducted our empirical experiments using the C5 and G4 EC2 (Elastic Compute Cloud) instances, designed for compute-intensive workloads and GPU computing respectively. In the us-east-2 region, the on-demand pricing of the AWS c5d.18xlarge EC2 instance, with 72 vCPUs and 144GB memory, is $3.888 per hour. The pricing for a g4dn.4xlarge, with single GPU, 16 vCPUs and 64GB memory, is $1.204 per hour [36]. The ratio of the costs of CPU vs. GPU is the time ratio 2551.8/88.9 multiplied by the pricing ratio 3.888/1.204 which works out to be 92.7-fold cheaper when the GPU instance is used for basecalling. These cost estimates are based on single samples.

## Conclusions

A potential advantage of long-read sequencing is lower costs in comparison to standard NGS. We used low-cost Flongles to detect the PML-RARA and BCR-ABL1 fusions. In this work, we demonstrate the ability to detect fusions on nanopores devices that read DNA at speeds faster than 1 nt/µs. We present interactive software tools that not only make analyses of Nanopore data accessible to biomedical and clinical scientists, but also efficient and economical through the use of GPU computing. Most importantly, we have illustrated the applicability of our workflow for the analysis of cell line data as part of a rapid, cost effective assay to detect fusions. Using an intuitive graphical interface, our workflow integrates the processing of raw nanopore reads, with the visualization steps that are used to interpret the results. This provides the capability to identify and confirm pathognomonic fusion genes such as BCR-ABL1 in CML or PML-RARA in APL. This methodology can potentially both define specific breakpoints important in treating and tracking a patients’ specific disease, but also allow multiplex phasing to identify and follow multiple mutations in the same patient. Future directions include optimizing the assay for faster library preparation, sequencing times, and analysis to enable fusion detection to less than a day. Improvements to turnaround time in the laboratory combined with an accessible, efficient informatics workflow that enables most molecular technologists and pathologists to implement long-read sequencing into current clinical pathology workflows will advance the field of molecular pathology beyond what is currently possible with NGS. These small portable, low-cost devices, together with integrated bioinformatics support, will allow for rapid diagnostics to assist in point-of-care clinical decision making.

## Availability and requirements

**Project name**: Nanopore-GPU.

**Project home page**: All code is publicly available and distributed under a custom academic license at https://github.com/BioDepot/nanopore-gpu. A public AMI (ami-0ecb1effab7fcfaa3) is provided with all necessary drivers and software pre-installed.

**Operating system(s)**: Platform independent.

**Programming language**: Python.

**Other requirements**: Docker.

**License**: Custom non-commercial license.

**Any restrictions to use by non-academics**: The software is used solely for noncommercial purposes.

## Supplementary Information

**Additional file 1.**

## Data Availability

Code and data for the workflows are available in the Github repository https://github.com/BioDepot/nanopore-gpu. The cell line Nanopore data are included in the workflow, with direct links from the README page. We have also created a demo video on Youtube https://youtu.be/yPhBKjdi8gY.

## Abbreviations

AMI: Amazon Machine Image
AML: acute myeloid leukemia
APL: acute promyelocytic leukemia
AWS: Amazon Web Services
Bwb: Biodepot-workflow-builder
CUDA: Compute Unified Device Architecture
EC2: Elastic Compute Cloud
GPU: Graphics Processing Unit
IGV: Integrated Genome Viewer
NGS: Next-generation sequencing
ONT: Oxford Nanopore Technologies
