# Supplementary material for: A graphical, interactive and GPU-enabled workflow to process long-read sequencing data
Source: BMC Genomics. 2021 Aug 23;22:626. doi: 10.1186/s12864-021-07927-1 (PMC8381503; doi:10.1186/s12864-021-07927-1)
Supplement: Supplementary file 1 — Additional file 1. [file 12864_2021_7927_MOESM1_ESM.docx]

**SUPPLEMENTARY INFORMATION**

**Additional file 1. Figure S1.** IGV viewer alignment on the CBFB and MYH11 genes of the reads obtained with a nanopore flow cell sequence for the cell line ME1 (panel A) and on KMT2A and AFF1 for MV4;11 (panel B). ME1 carries the inv(16) resulting in CBFB-MYH11 fusion and MV4;11 carries t(4;11) with KMT2A-AFF1 fusion. Libraries were generated from DNA with a PCR-free enrichment protocol using CRISPR guides targeting CBFB and MYH11 in the first experiment and KMT2A and AFF1 in the second one. Reads that span two genes are colored.


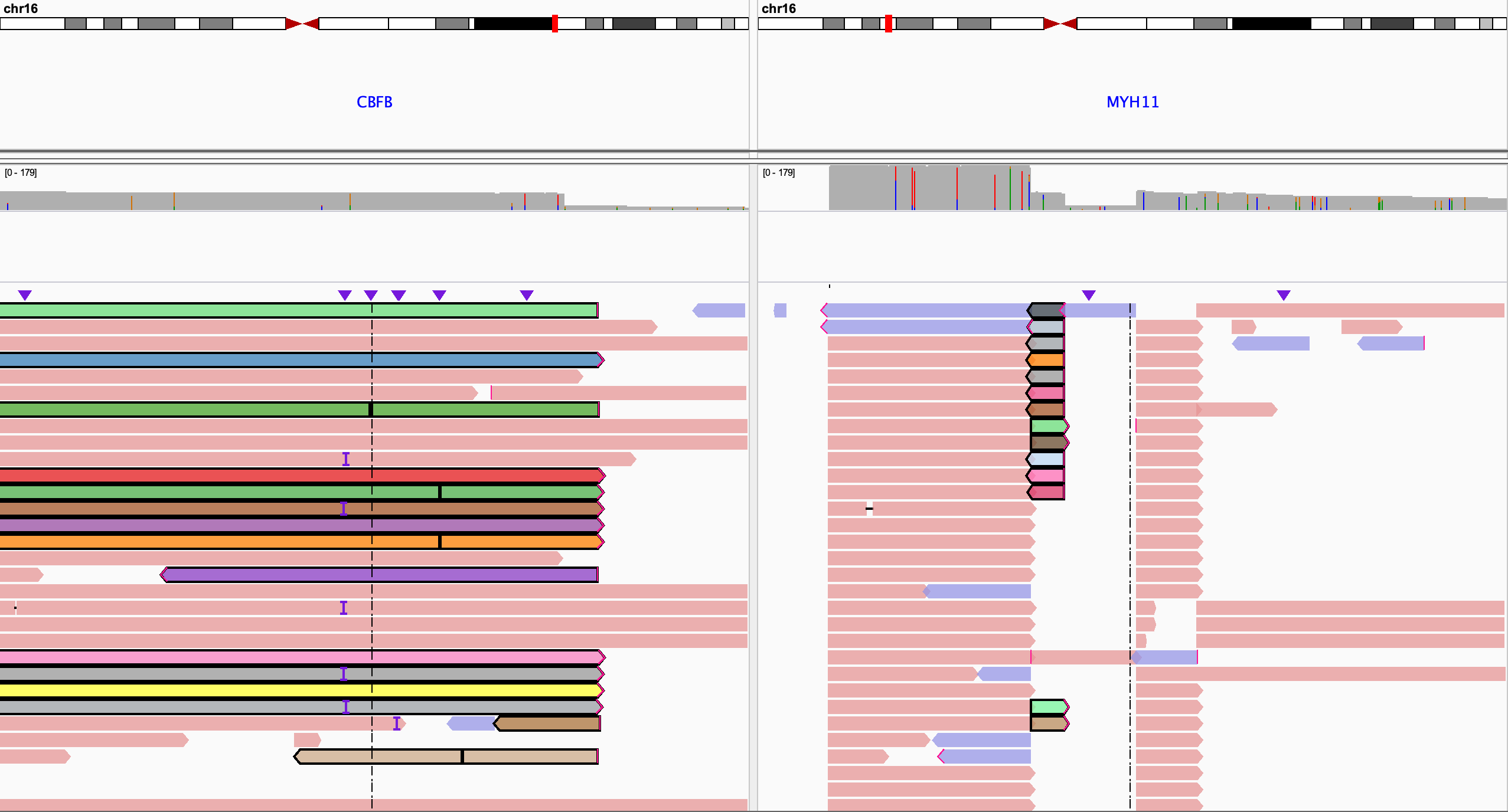

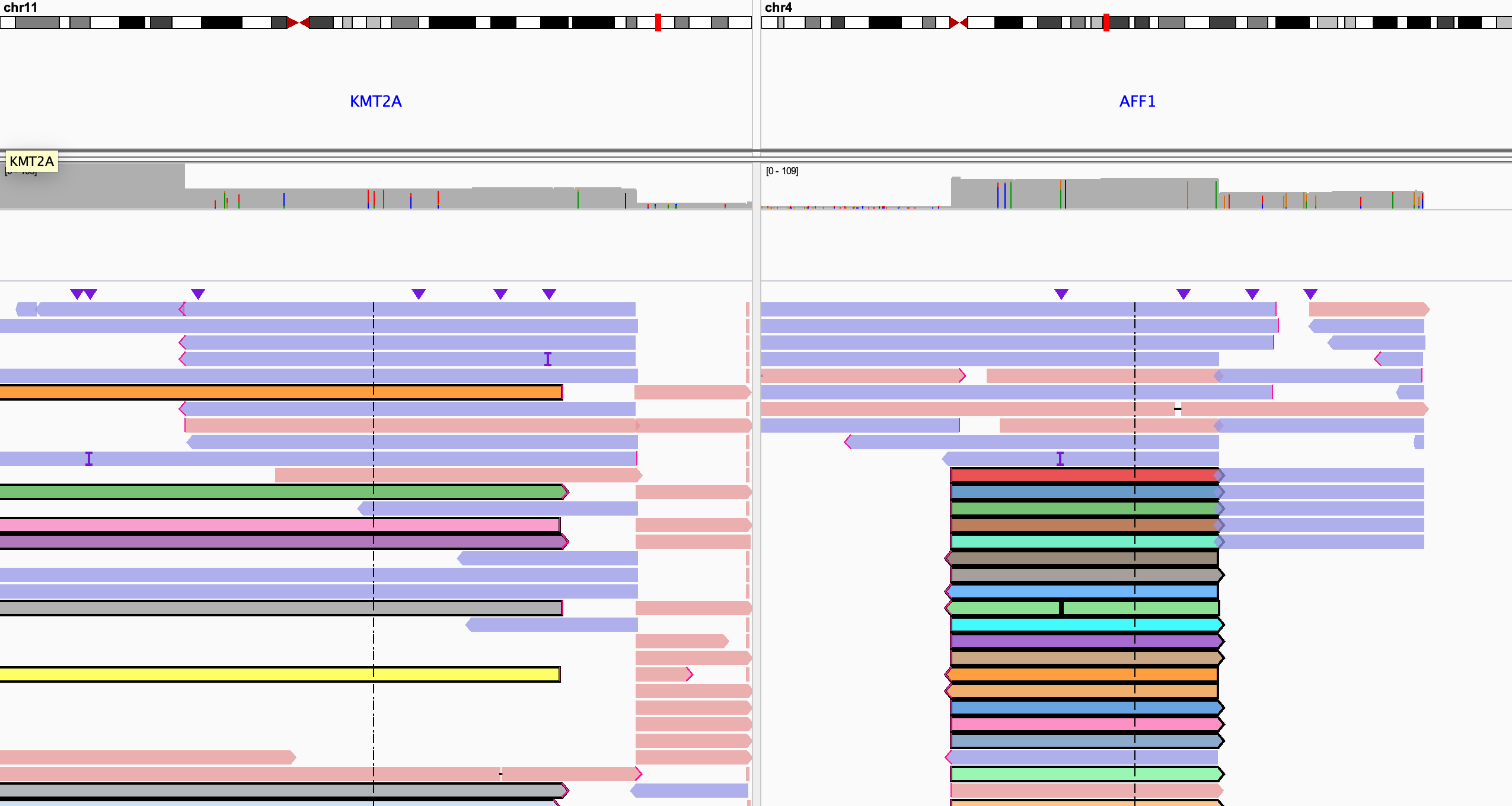


A

B
